# SLC35A2 expression is associated with HER2 expression in breast cancer

**DOI:** 10.1007/s12672-024-00978-2

**Published:** 2024-04-19

**Authors:** Yiran Wang, Xiaobo Peng, Meihong Wu, Bin Wang, Tianran Chen, Xianbao Zhan

**Affiliations:** Department of Oncology, Shanghai Changhai Hospital, Naval Medical University, Shanghai, 200433 China

**Keywords:** SLC35A2, Breast cancer, Immunohistochemistry, HER2, Prognosis

## Abstract

The role of SLC35A2 in breast cancer remains poorly understood, with limited available information on its significance. This study aimed to investigate the expression of SLC35A2 and clinicopathological variables in breast cancer patients. Immunohistochemical analysis of SLC35A2 protein was conductedon 40 adjacent non-neoplastic tissues and 320 breast cancer tissues. The study also assesed the association between SLC35A2 expression and breast cancer clinicopathological features of breast cancer, as well as its impact on overall survival. In comparison to adjacent non-neoplastic tissues, a significantly higher expression of SLC35A2 was observed in breast cancer tissues (*P* = 0.020), and this expression was found to be independently correlated with HER2 positivity (*P* = 0.001). Survival analysis indicated that patients with low SLC35A2 expression had a more favorable prognosis in HER2-positive subtype breast cancer (P = 0.017). These results suggest that SLC35A2 is overexpressed in breast cancer tissues compared to adjacent non-neoplastic tissues and may serve as a potential prognostic marker for HER2-positive subtype breast cancer. Furthermore, breast cancer patients with the HER2 positive subtype who exhibited decreased levels of SLC35A2 expression demonstrated improved long-term prognostic outcomes.

## Introduction

Breast cancer is the malignant cancer among all female malignant tumors worldwide with the highest incidence [1]. In the United States, incidence of breast cancer continued a slow increase for female during 2014 through 2018, and it is estimated that there will be 290, 560 female breast cancer patients, accounting for nearly one third of all newly diagnosed cases [2]. Meanwhile, breast cancer accounted for 16.72% of all new cases with more than 306,000 diagnoses in female of China in 2016 [3]. Current treatment options for breast cancer have improved, yet remain suboptimal for many patients. The development of novel anticancer drugs relies on the discovery of new mechanisms for breast cancer. The pathogenesis of lung cancer possibly related to disfunction nucleotide sugar transportation [4], but it is remained unexplored in breast cancer. Therefore, exploring the association between clinicopathological factors and key proteins is crucial for investigating the mechanism and advancing the development of novel therapeutics for breast cancer.

Nucleotide sugar transporters are one of the endoplasmic reticulum and Golgi-resident members of the solute carrier 35 (SLC35) family [5], and it play the role of suppling substrates for glycosylation by exchanging endonucleotide monophosphates for cytosolic nucleotide sugars [6]. Gene mutation of nucleotide sugar transporters are known to associate into various types of complexes, such as congenital disorder of glycosylation type IIm (CDG2M) [7]. Additionally, nucleotide sugar transporters are primarily involved in the metabolism of glucose, which is intensely utilized by cancer [8]. And a better understanding of this membrane protein family has become critical for cancer metabolic research. Although evidence has shown that SLC35A2 may play a role in tumorigenesis [9], its expression and clinicopathological significance in breast cancer are still unclear. In order to have a better understanding of SLC35A2 in breast cancer, we investigated its expression profile, clinical significance and the impact of its expression on survival.

This study aims to explore the relationship between SLC35A2 expression and clinical parameters of breast cancer, and the influence of SLC35A2 expression on the prognosis of breast cancer patients.

## Materials and methods

### Clinical data and tissue samples

A total of 320 tissue specimens were collected from primary early-stage (stage I–III) breast cancer patients who underwent the operation from February 2002 to March 2010 in Shanghai Changhai Hospital. Clinical data were extracted from patients’ medical documents, including diagnosis age, TNM staging (8th edition of AJCC cancer staging), pathological type, subtype (St. Gallen), histological grading and clinical outcomes. The median follow-up time was 111 months, and overall survival (OS) was defined as the time from the date of the primary surgery to the time (in months) of death from breast cancer. All invasive carcinomas have no special type. Breast cancer molecular subtypes were defined by estrogen receptor (ER), progesterone receptor (PR) and the human epidermal growth factor 2 (HER2) expression. Breast cancer subtypes were defined as luminal A-like (ER + PR + HER2 − low Ki67), luminal B-like (ER + PR − HER2 − or ER + PR + /PR − HER2 + or high Ki67), HER2-positive (ER − PR − HER2 +) and triple negative (ER − PR − HER2 −). The positive cut-off value of ER is set to ≥ 1% of cells with positive staining. Meanwhile, and cutoff for high expression of PR was set at ≥ 20% of cells with positive staining. HercepTest™ were used to assess HER-2 status according to the manufacturer’s instructions. HER-2 high expression was defined as an IHC score of 3 + for more than 10% of invasive tumor cells. FISH analysis was performed when the tumors showed 2 + score by IHC. HER-2 expression is considered to be amplified, if the ratio of HER-2 signal to chromosome 17 signal is greater than 2.0. HER2-low is currently defined as invasive breast carcinoma with HER2 immunohistochemistry (IHC) score of 1 + or 2 + with a negative in situ hybridization (ISH) assay. Ki-67 index is classified as low if it is less than 20%. In addition, forty paracancerous tissue samples were collected from breast cancer surgery as control group.

SLC35A2 immunohistochemical staining was performed in 320 tumor tissues and 40 adjacent non-tumor tissues. The study was approved by the Ethics Committee of Medicine at Shanghai Changhai Hospital. And all methods conform to standards of the Declaration of Helsinki. The study obtained the written informed consent of all patients.

### Immunohistochemistry and tissue microarray

Tissue microarrays (TMA) were derived from cancer and adjacent tumor tissues. Tissues were stored in 10% neutral formalin for block preparation. The tissue arraying instrument was used to make TMA blocks. The cylindrical tissue strips (1.5 mm in diameter) were extracted from the tumor tissue center or the adjacent tissue center, and then arranged into blank recipient paraffin blocks. Immunohistochemical staining was performed on 4-μm sections of TMA with primary SLC35A2 antibody (1:200; catno. PA5-57514; Thermo Fisher Scientific, Waltham, MA, USA). Primary antibody rabbit immunoglobulin G was used for a negative control with the same dilution. High‐throughput automated scanner was used to digitally scan immunohistochemical staining slides at 40 × magnification.

Two pathologists independently scored the SLC35A2 staining of each specimen and repeated it three times. The percentage of positive staining cells and staining intensity of each tissue sample were evaluated. The final SLC35A2 expression score can be obtained by multiplying the staining intensity score (0, negative; 1, weak; 2, moderate; 3, strong staining) by the staining area percentage score (0, negative; 1, < 10%; 2, 11–50%; 3, 51–80%; and 4, > 80%). The specimens were divided into two groups according to the total score (≤ 5, low expression; > 5, high expression) [10, 11].

### Gene correlation analysis

All raw data were obtained from the TCGA database. And GEPIA (The web address is http://gepia.cancer-pku.cn/) was applied to generate Fig. 3.

### Statistical analyses

The IBM SPSS Statistics (for Windows, version 21.0) was used for statistical analyses. The expression of SLC35A2 in adjacent non tumor tissues and cancer tissues were compared using Chi-square test or Fisher´s exact test. The correlations between clinicopathological factors were also confirmed by logistic regression analysis. The Kaplan–Meier survival curves were used to assess the effect of SLC35A2 expression and OS in various molecular subtypes. The Kaplan–Meier survival curves were plotted with log-rank test. In this study, differences were considered statistically significant when P < 0.05.

## Results

### Analysis of clinical data

**Fig. 1:**
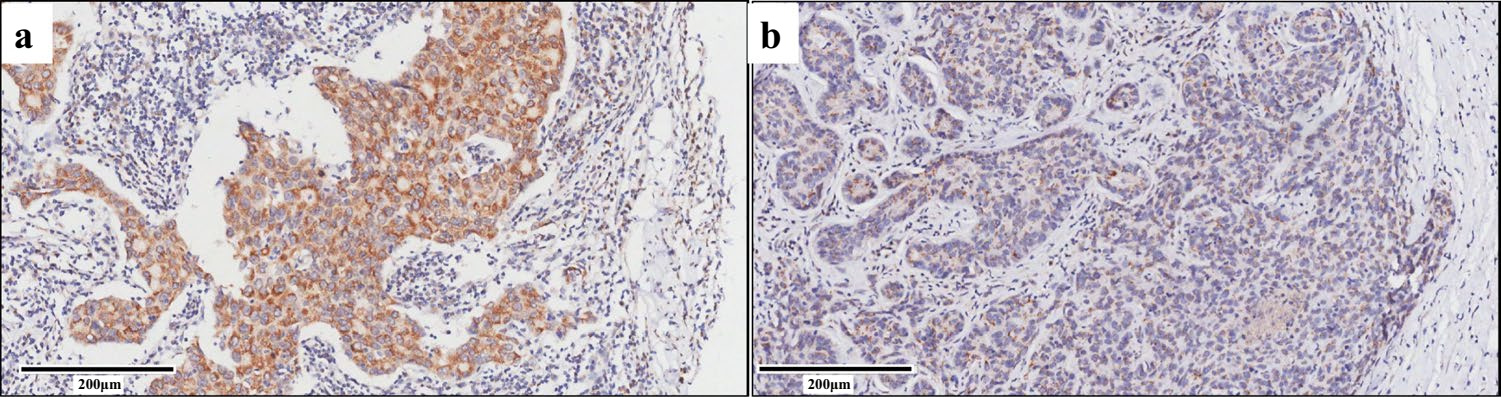
Representative images from IHC analysis show SLC35A2 expression levels in cancer tissues. **a** High expression in breast cancer tissues. Staining intensity score 3 multiplied staining area percentage score 4 (> 80%) is expression score 12. **b** Low expression in breast cancer tissues. Staining intensity score 1 multiplied staining area percentage score 2 (11–50%) is expression score 2. The magnification is 10 × and the scale bar is 200 μm

The immunohistochemical staining of SLC35A2 was observed in 320 tumor tissue samples and 40 paracancerous tissue samples (Fig. 1). A total of 64 (20%) were non- invasive ductal carcinoma (IDC), and 256 (80.0%) were IDC among these 320 tumor tissue samples. In terms of molecular subtypes, there were 34 (10.6%) for luminal A, 163 (50.9%) for luminal B, 71 (22.2%) for Her-2 positive and 52 (16.3%) for triple negative subtype breast cancer. There were 210 (65.6%) for grade I–II and 110 (34.4%) for III as for histological grades. Furthermore, there were 77 (24.1%) for TNM stage I, 161 (50.3%) for TNM stage II, and 82 (25.6%) for TNM stage III.

### Expression of SLC35A2 in tumor tissues and paracancerous tissues

SLC35A2 is mainly expressed in cytoplasm. Overall, high expression of SLC35A2 was found in 37.5% (15/40) adjacent noncancerous tissues and 56.9% (182/320) cancer tissues. Breast cancer tissues were statistically significantly more likely to express SLC35A2 than adjacent noncancerous tissues (P = 0.020, Table 1).

**Table 1:** Expression of SLC35A2 in breast carcinoma tissues and adjacent non-neoplastic tissues

Characteristics	SLC35A2 Expression		Total	χ^2^	*P*
	High (%)	Low (%)			
Breast carcinoma tissues	182 (56.9)	138 (43.1)	320		
Adjacent non-neoplastic tissues	15 (37.5)	25 (62.5)	40	5.387	0.020

### Correlation among clinical pathological characteristics and SLC35A2 expression

The clinical factors include the following: diagnosis age, pathology types, TNM stage, molecular subtypes, histological grade, ER, PR, HER2 and Ki-67 index. A significant correlation was found between SLC35A2 expression and HER2 status, but not between SLC35A2 expression and diagnosis age, TNM stage, molecular subtypes, ER, PR, histological grade, Ki-67 index and pathology types. The expression rate of SLC35A2 was 43.2% (19/44) in HER2 negative group, 48.2% (55/114) in HER2 low group and 66.7% (108/162) in HER2 highe group. The difference was statistically significant (P = 0.001, Table 2).

**Table 2:** Correlation between SLC35A2 expression and clinic pathological factors in 320 breast cancer tissues

Characteristics	SLC35A2 Expression		Total	χ^2^	*P*
	High (%)	Low (%)			
Age at diagnosis (years)					
≤ 55	105 (57.4)	78 (42.6)	183		
> 55	77 (56.2)	60 (43.8)	137	0.044	0.834
Pathology type					
IDC	144 (56.3)	112 (43.7)	256		
Non-IDC	38 (59.4)	26 (40.6)	64	0.204	0.652
Histological grade					
I–II	116 (55.2)	94 (44.8)	210		
III	66 (60.0)	44 (40.0)	110	0.667	0.414
ER					
Positive	106 (53.3)	93 (46.7)	199		
Negative	76 (62.8)	45 (37.2)	121	2.794	0.095
PR					
High ≥ 20%	75 (53.6)	65 (46.4)	140		
Low < 20%	90 (60.0)	60 (40.0)	150		
Negative	17 (56.7)	13 (43.3)	30	1.221	0.543
HER2					
Negative	19 (43.2)	25 (56.8)	44		
Low	55 (48.2)	59 (51.8)	114		
High	108 (66.7)	54 (33.3)	162	13.157	0.001
Ki-67					
≥ 20%	138 (58.7)	97 (41.3)	235		
< 20%	44 (51.8)	41 (48.2)	85	1.232	0.267
Intrinsic subtypes					
Luminal A	15 (44.1)	19 (55.9)	34		
Luminal B	90 (55.2)	73 (44.8)	163		
Her2 positive(non-luminal)	52 (73.2)	19 (26.8)	71		
Triple negative	25 (48.1)	27 (51.9)	52	11.832	0.008
Tumor size					
≤ 2 cm	69 (57.5)	51 (42.5)	120		
2–5 cm	97 (56.4)	75 (43.6)	172		
> 5 cm	16 (57.1)	12 (42.9)	28	0.036	0.982
Number of lymph metastases					
0	97 (56.4)	75 (43.6)	172		
1–3	44 (57.9)	32 (42.1)	76		
4–9	21 (60.0)	14 (40.0)	35		
≥ 10	20 (54.1)	17 (45.9)	37	0.308	0.959
TNM stage					
I	42 (54.5)	35 (45.5)	77		
II	93 (57.8)	68 (42.2)	161		
III	47 (57.3)	35 (42.7)	82	0.229	0.892
Menopausal status					
Premenopausal	75 (56.4)	58 (43.6)	133		
Postmenopausal	107 (57.2)	80 (42.8)	187	0.022	0.883

*IDC* invasive ductal carcinoma, *ER* estrogen receptor, *PR* progesterone receptor, *HER2* human epidermal growth factor receptor 2

The ER status (*P* = 0.095) and HER2 status (*P* = 0.001) were included in the logistic regression model analysis (excluding the molecular subtypes, because their effects were affected by ER status, PR status and HER2 status). Multivariate analysis confirmed that HER2 high expression (*P* = 0.006) was closely related to the high expression of SLC35A2, but not HER2 low expression (*P* = 0.487) (Table 3).

**Table 3:** Multivariate analyses by logistic regression analysis

Dependent variable	Independent variable	SE	*P* value	OR	95% CI
SLC35A2 high expression	ER positive	0.244	0.234	0.748	0.464–1.207
	HER2 low	0.361	0.487	1.285	0.634–2.604
	HER2 high	0.348	0.006	2.608	1.319–5.157

### Survival analysis

**Fig. 2:**
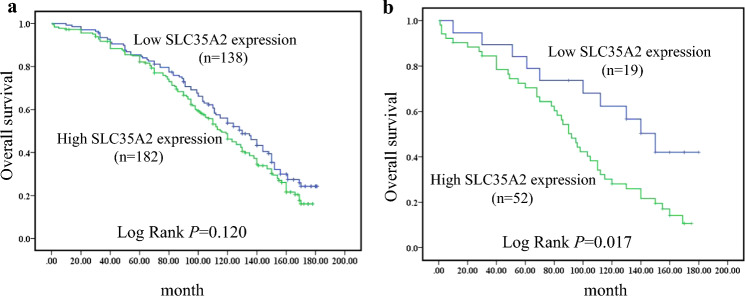
Kaplan–Meier curve of overall survival in relation to SLC35A2 protein expression in breast cancer. **a** Breast cancer, **b** HER2 subtype breast cancer

The follow-up was conducted from February 2002 to July 2017. When all subtypes of breast cancer were analyzed together, SLC35A2 expression was not related to OS (P = 0.120) (Table 4). However, for HER2 positive subtype patients, the median survival time of the low SLC35A2 expression group was 128.6 months (SE: 13.17, 95%CI: 102.25–153.86) and SLC35A2 overexpression group was 93.01 months (SE: 7.408, 95% CI: 78.49–107.53) (P = 0.017). Additionally, the 5-year and 10-year survival rates were 69.2% and 28.8% respectively in the group with high SLC35A2 expression, and 84.2% and 57.9% respectively in the group with low SLC35A2 expression. Figure 2 showed Kaplan–Meier survival curves.

**Table 4:** Relationship between overall survival and SLC35A2 in patients with different subtypes of breast cancer

Analyses for SLC35A2	Log rank	*P* value
All types as total	2.419	0.120
Luminal A	0.019	0.891
Luminal B	0.576	0.448
HER2	5.705	0.017
Triple negative	0.608	0.435

## Discussion

The solute carrier family SLC35 includes several nucleotide sugar transporters and the solute carrier family SLC35 is divided into 7 subfamilies (from SLC35A to SLC35G) [12]. The SLC35A subfamily includes 5 paralogous (from SLC35A1 to SLC35A5) [13]. SLC35A2 plays a role in glycogen metabolism and encodes the X-linked transporter that imports uridine diphosphate (UDP)-galactose into the Golgi and the endoplasmic reticulum for glycosylation [14]. Gene mutations of SLC35A2 generate a spectrum of phenotypes [15]. Especially, novel gene variants of SLC35A2 and congenital disorders of glycosylation (CDG) is gradually receiving greater focus [16, 17]. To some extent, abnormal protein expression of SLC35A2 were somewhat more prevalent than gene mutations. Abnormal protein expression of SLC35A2 was more prevalent than gene mutations. Not only should we pay attention to abnormalities at the gene level, but we should not ignore the effects of abnormal protein expression in individuals with normal genes [18, 19].

**Fig. 3:**
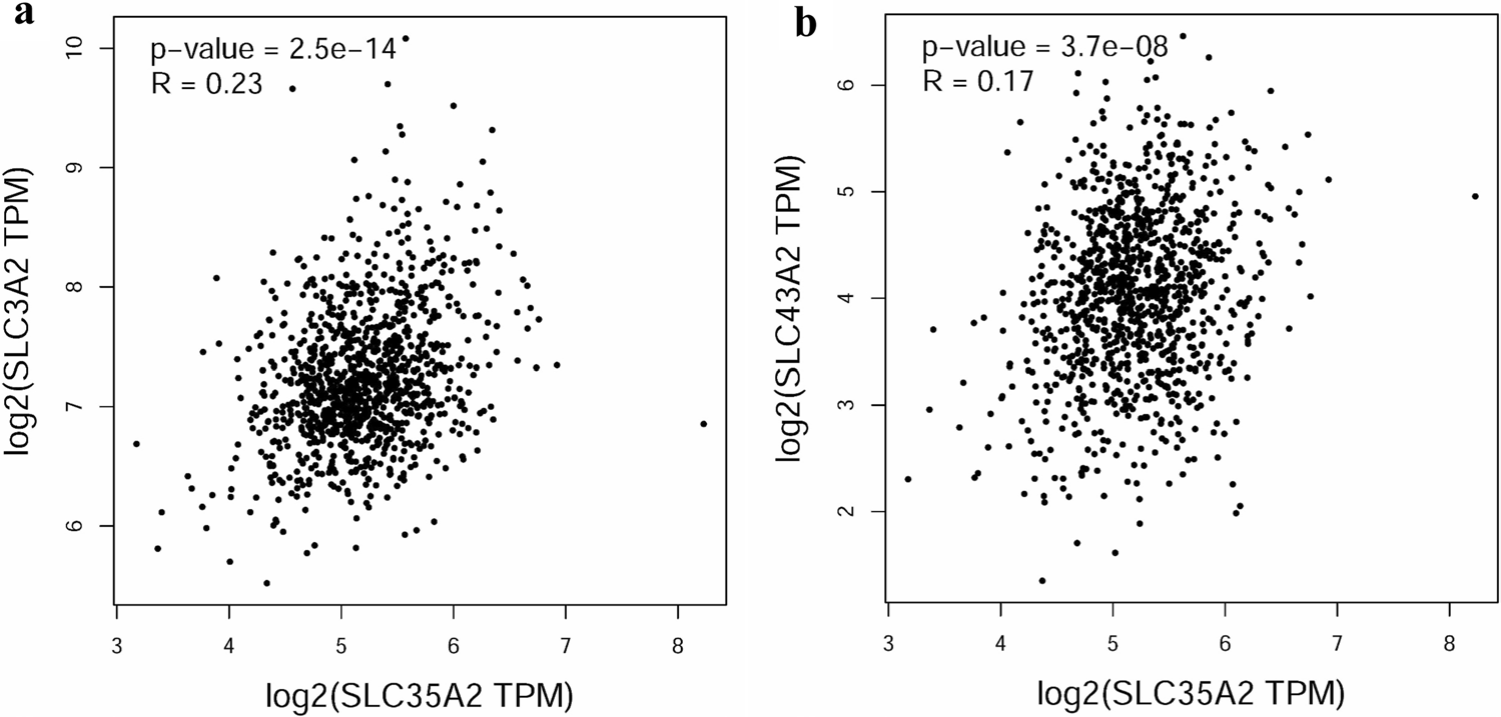
SLC35A2 expression positively correlates with SLC3A2 and SLC43A2 based on TCGA database. **a** SLC3A2, **b** SLC43A2

SLC35A2 plays an important role in regulating glucose metabolism, and a large number of tumor cells have high metabolic activity [20]. SLC35A2 may contribute to the growth of tumor, but particular functions of SLC35A2 in breast cancer are still unclear regardless of awareness that they play critical roles in tumorigenesis and progression [21]. The family of SLC proteins regulate multiple metabolic and signaling pathways. Some studies explored the role of solute carrier family SLC35 in various cancers [22, 23]. SLC3A2 is upregulated in several cancers, such as human osteosarcoma, gastric cancer and breast cancer, and promotes tumor growth through ferroptosis signaling pathway [24, 25]. Their study lacked detailed analysis for breast cancer, such as the relationship between different clinical characteristics of breast cancer patients and SLC35A2, which is complementary to our study. Meanwhile, SLC43A2 alters T cell methionine metabolism and histone methylation in cancer [26]. Based on the information from TCGA database, SLC35A2 expression positively correlates with SLC3A2 expression in human breast cancer (Fig. 3a). Therefore, the relationship between SLC35A2 and ferroptosis deserves further exploration. Additionally, SLC43A2 and SLC35A2 are positively correlated according to TCGA database (Fig. 3b), hence the relationship between SLC35A2 and tumor immunity is worth exploring.

Our results showed that there was statistically positive correlation between HER2 and SLC35A2. HER2 is overexpressed in about 20% of breast cancer and is a member of the human epidermal growth factor transmembrane receptor family [27]. HER2-positive breast cancers have high rates of metastasis and recurrence, and are among the most dangerous pathological types of breast cancer [28]. High HER2 levels are associated with enhanced activation of the PI3K/AKT and MAPK pathways of cancer, leading to increased proliferation and survival [29], and it is known that SLC35A2 can also promotes cell metabolism. It is necessary to explore whether there is an upstream and downstream relationship between HER2 and SLC35A2.

This study proposes new ideas and intervention measures for the treatment of breast cancer. HER2-targeted therapy was recommended for patients with HER2‐high expression. Despite many HER2 positive breast cancer patients benefiting from anti-HER2 therapy, some patients develop resistance and ultimately experience disease progression [30]. Kaplan Meier survival curve suggested that HER2 positive subtype breast cancer patients have poor prognosis with high SLC35A2 expression. Drug resistance of anti-HER2 therapy may related to poor prognosis. The relationship between SLC35A2 and anti-HER2 therapy resistance will be more specifically addressed in future studies. The proportion of primary drug resistance will be analyzed between patients with high and low SLC35A2 expression. Understanding the drug resistance of anti-HER2 therapy may reveal novel personalized and more efficient therapeutic strategies. Anti-SLC35A2 therapy maybe an option to overcome resistance to HER2 blockade for HER2 positive subtype breast cancer patients. However, for HER2-positive patients, there is a difference in SLC35A2 expression between patients with high and low HER2 expression, which warrants further exploration.

Nowadays, new therapeutic concepts appeared to improve the treatment of breast cancer. Based on the IHC and FISH/SISH, HER2 low expression was defined as HER2 1 + and 2 + with lack of HER2 amplification. Anti-HER-2 antibody–drug conjugates (ADCs) have shown notable clinical benefits in breast cancer patients with HER2 low expression. Results suggested that HER2-low breast cancer patients might still benefit from anti-HER2 treatments, but the combination of molecular-targeted therapy and chemotherapy is needed. For HER2 low expression patients, it deserves to explore whether combination therapy of anti-HER2 and anti-SLC35A2 is effective.

The primary limitation of this study included the lack of treatment information. In this study, patients were included who underwent surgery between 2002 and 2010. In view of the high cost of Herceptin and non-standardized treatment, not all eligible HER2 positive patients received postoperative adjuvant HER2 targeted therapy before 2010. Therefore, it is unclear whether the overall survival of HER2 patients was disturbed by nonstandard treatment. The relationship between prognosis and different treatment schemes in breast cancer should be explored in future. Moreover, the possible selective bias due to the inclusion of only postoperative patients. Additionally, we could not analyze the relationship between PFS (progression-free survival) and SLC35A2 expression because of a lack of PFS data. Although this study had some limitations, the relationship between the expression of SLC35A2 and clinicopathological parameters and prognosis of breast cancer has been verified.

## Conclusions

Compared with adjacent non-neoplastic tissues, high SLC35A2 expression were observed in breast cancer tissues significantly (P = 0.020). And it was correlative with HER2 positive independently (P = 0.001). It was concluded that patients with low SLC35A2 expression had a better prognosis for HER2-positive subtype breast cancer by survival analysis (P = 0.017). Our findings demonstrated that SLC35A2 was highly expressed in breast cancer tissues compared with adjacent non-neoplastic tissues. Moreover, expression of SLC35A2 may serve as a novel prognostic marker for HER2 positive subtype breast cancer.

## Data Availability

The data used to support the findings of this study are available from the corresponding author upon request.
